# Operating list composition and surgical performance

**DOI:** 10.1002/bjs.10804

**Published:** 2018-03-20

**Authors:** T. W. Pike, F. Mushtaq, R. P. Mann, P. Chambers, G. Hall, J. E. Tomlinson, R. Mir, R. M. Wilkie, M. Mon‐Williams, J. P. A. Lodge

**Affiliations:** ^1^ Faculty of Medicine and Health University of Leeds Leeds UK; ^2^ School of Mathematics University of Leeds Leeds UK; ^3^ Leeds Institute for Data Analytics, University of Leeds Leeds UK; ^4^ Leeds Teaching Hospitals NHS Trust Leeds UK; ^5^ Spire Healthcare, Spire Leeds Hospital Leeds UK; ^6^ Department of Orthopaedics Sheffield Teaching Hospitals Sheffield UK; ^7^ Department of Medical Education Sheffield University Sheffield UK

## Abstract

**Background:**

Recent reviews suggest that the way in which surgeons prepare for a procedure (warm up) can affect performance. Operating lists present a natural experiment to explore this phenomenon. The aim was to use a routinely collected large data set on surgical procedures to understand the relationship between case list order and operative performance.

**Method:**

Theatre lists involving the 35 procedures performed most frequently by senior surgeons across 38 private hospitals in the UK over 26 months were examined. A linear mixed‐effects model and matched analysis were used to estimate the impact of list order and the cost of switching between procedures on a list while controlling for key prognosticators. The influence of procedure method (open versus minimally invasive) and complexity was also explored.

**Results:**

The linear mixed‐effects model included 255 757 procedures, and the matched analysis 48 632 pairs of procedures. Repeating the same procedure in a list resulted in an overall time saving of 0·98 per cent for each increase in list position. Switching between procedures increased the duration by an average of 6·48 per cent. The overall reduction in operating time from completing the second procedure straight after the first was 6·18 per cent. This pattern of results was consistent across procedure method and complexity.

**Conclusion:**

There is a robust relationship between operating list composition and surgical performance (indexed by duration of operation). An evidence‐based approach to structuring a theatre list could reduce the total operating time.

## Abstract 2

Science behind list structure

## Introduction

There is the potential for large routinely collected clinical data sets to improve healthcare delivery^1^, ^2^, ^3^. More specifically, systematic and statistical examination of operating records could provide novel insights into surgical practice^4^, ^5^, ^6^, ^7^, ^8^. Given that operating theatres are one of the costliest elements of a hospital^9^, these advances present important new opportunities for understanding how to improve surgical quality and efficiency^10^, ^11^.

Examining routinely collected data from the operating theatre allows the creation of natural experiments (where exposure to the event of interest has not been manipulated experimentally^12^, ^13^). This allows the description, characterization and prediction of organizational, administrative and human factor‐related behaviours.

Previous studies have implicated a number of factors that influence the amount of time it takes for a surgeon to perform a procedure. Beyond the surgeon's level of skill^14^, ^15^, research has demonstrated the influence of external drivers on procedure duration, including: patient characteristics^15^ (for example age^14^, ^16^, co‐morbidities such as BMI^17^, ^18^, ^19^), the surgical team^20^, ^21^, hospital size^22^ and case mix^23^. Recent reviews^24^, ^25^ have suggested that the way in which surgeons prepare for an operation can also affect performance, with some preparation techniques resulting in shorter operating times. The majority of these studies involved simulated contexts^26^, ^27^ and conclusions about best practice for preparation in the real world remain unclear.

Operating lists present a natural experiment to test the hypothesis that surgeons will warm up progressively through practice, and that such benefits will be ameliorated when surgeons switch procedures in a theatre list. Examination of the impact of list order may also yield important information about service organization efficiency^28^. Anecdotal evidence suggests that some surgeons schedule what is perceived to be their most difficult operation first. This may be sensible but, to date, there is no evidence base to support such practice. Similarly, there has been no investigation of whether a list should be unimodal (1 procedure and/or method, for example open or minimally invasive) or multimodal in composition. Analysis of routinely collected information on surgical procedures presents an opportunity to move away from using clinical intuition and experience‐driven decisions to data‐driven decision‐making^29^, ^30^, ^31^, ^32^. To this end, the aim of this study was to examine the effect of operating list order on duration of operation in procedures performed across all 38 Spire Healthcare hospitals, one of the largest providers of private healthcare in the UK.

## Methods

This study received ethical approval from the Spire Healthcare Research Ethics Committee. Data were collated from Spire Healthcare's electronic patient record system (SAP SE, Walldorf, Germany) across all 38 UK hospitals. To practice in a private hospital in the UK, a surgeon must be on the General Medical Council's specialist register and hold, or have held in the past 5 years, a substantive consultant post within the National Health Service (NHS) or a Defence Medical Services hospital. Consequently, all procedures in these hospitals were performed by experienced consultant surgeons, assisted if appropriate by trainees. Patient demographics, procedural/operative information, prognosticators of operative outcome (ASA physical status grade^33^) and duration of hospital stay were included in the data set. Age was divided into groups, to allow adequate anonymization of data (18 or less, 19–24, 25–34, 35–44, 45–54, 55–64, 65–75, over 75 years). No additional information (such as sex or co‐morbidity) was available to the research team.

The 35 most frequently observed operations in the data set were the primary focus of investigation. No restriction was placed on the surgical subspecialty, type of procedure performed, or techniques used by the operating surgeon to perform the procedure. The collated data were parsed to allow further analysis; individual surgeon's operating lists were identified, and any list that contained one of the most frequent 35 operations was included in the data set (98 291 theatre lists). Any other procedure performed during one of those lists was also included in the data set (255 757 procedures in total).

Component operations were allocated absolute and procedure‐specific order numbers. The absolute list number refers to the number of procedures performed by the operating surgeon on the list, whereas the procedure‐specific list number is the number of times a certain procedure has been performed by the surgeon on a list. All cases that involved a change from the previous procedure were coded as a switch, because they involved some form of task switching (*Table* 1).

**Table 1:** Illustration of absolute and procedure‐specific list number and switch classification

Procedure	Absolute list no.	Procedure‐specific list no.	Switch
Laparoscopic cholecystectomy	1	1	n.a.
Open inguinal hernia repair	2	1	Yes
Laparoscopic cholecystectomy	3	2	Yes
Laparoscopic cholecystectomy	4	3	No
Open inguinal hernia repair	5	2	Yes

n.a., Not applicable.

Procedures were also classified by method (open or minimally invasive surgery) and complexity, in accordance with the AXA Specalist Procedure Codes, which are used to grade the magnitude of surgical procedures in UK independent hospitals (*Table* S1, supporting information)^34^.

**Figure 1:**
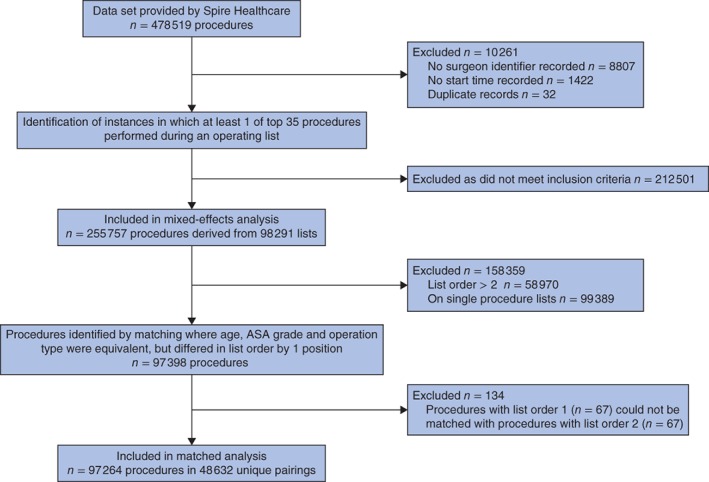
Flow chart illustrating how sample sizes were determined for the linear‐mixed effects and matched analyses from the original data set

From the original data set comprising 478 519 individual procedures, 8807 were excluded because they had no surgeon identifier associated with them and 1422 because no start time was recorded (*Fig*.  1). Thirty‐two duplicate records were also removed. Although such instances were relatively trivial to identify, a more difficult challenge in analysing routinely collected data lay in identifying cases where erroneous data might have been entered, for example the wrong start time or procedure type, or instances where missing data might have been due to the procedure ultimately being cancelled. All of these factors are likely to influence procedure order classification. This introduced noise, which it was reasoned would work against the hypothesis being tested (because the hypothesis suggests that the preceding operation (*n* – 1) affects the subsequent one; where data are missing, using *n* – 2 would make it more likely that the hypothesis would be rejected). Importantly, because of the statistical power afforded by a data set of this size, this noise in the data was tolerated rather than adjusting list order numbering, which would have required subjective inferences.

Duration of operation was employed as the primary outcome measure. Generally, this measure was defined as the time from skin incision to skin closure. In procedures where a skin incision is not made (such as endoscopic examination), the time taken for the procedure to be performed (defined as time from insertion to withdrawal of the endoscope for endoscopic examinations) was used. This measure was chosen because it is strongly correlated with surgeon performance, the focus of the study^35^, and previous studies^36^, ^37^, ^38^, ^39^, ^40^ have shown a relationship between this variable and clinical outcomes across a range of operations. In addition, duration of operation is recorded routinely in Spire Healthcare hospitals, and is not affected by loss of patients to follow‐up, unlike other measures of clinical outcome such as hospital death. Duration of hospital stay (in minutes) was investigated as a secondary outcome measure.

### Statistical analysis

Operating times are zero‐bound and present a skewed distribution^14^. Therefore, all analyses focused on changes in natural logarithmic operating time, which can be seen as equivalent to measuring proportional time changes for relatively small magnitudes. A model was created to capture the effects of absolute list order, procedure‐specific list order and switching acrosss the full range of list positions to understand the relationship between list composition and duration of operation. It was also reasoned that different operations might yield distinctly different patterns of results, and so the analysis was conducted at a procedure level to allow individual cases to be compared against the same types of procedure.

As the data set included information on factors known to correlate with postoperative outcomes (ASA grade^5^, ^6^, ^41^, ^42^ and age^43^, ^44^, ^45^), these potential confounders were controlled for. Different ages and ASA grades, along with different surgical procedures would imply different normal operating times; therefore, these baseline operating times were treated as random effects, shared by all operations of the same type, on the same age group and with the same ASA grade. In reality, operations on patients in similar age groups or with similar ASA grades will have similar baselines; for example, a 34‐year‐old patient with an ASA grade of II is more similar to a 40‐year‐old with an ASA grade of II than an 80‐year‐old with a grade of IV. However, assumptions were not made about the relationship between duration of operation and these factors. Instead, a statistically more conservative approach was adopted by assuming that these random effects were independent between pairs of operations (unless all 3 of these variables were identical). Restricted‐likelihood maximization via the Lme4 package^46^ was used to fit the linear mixed‐effects model in R (R Project for Statistical Computing, Vienna, Austria), and the effect size and probability values (α threshold of P < 0·050) estimated for the fixed effects of list order (absolute and procedure‐specific) and switching.

For a closer examination of the primary effects observed in the data, a form of matched analysis was subsequently performed on a subset of the data. This analysis was inspired by (but not identical to) a novel method for identifying causal relationships in natural experiments^47^. Here, the data were stratified into multiple sets of pairs by explicitly matching individuals who had the same age, ASA grade and operation type, but differed in list order by one position. Specifically, the data were first filtered by procedure type, then all cases that were ordered as procedures 1 and 2 were separated into different data frames (list order 1 and list order 2). All cases in list order 1 (presented in a randomly determined order) were examined to determine which elements of list order 2 had the same age group and ASA grade. If a case could be matched, this pair was included in the subsequent analysis and removed from the pool. In the event of multiple matches from list order 2 with list order 1, the computer program randomly selected one case for the pair and the non‐selected case(s) were returned to the pool for a possible future match. Each patient was paired to only one other individual, and only patients for whom a pair could be found were included. The matching process terminated when no more unique pairs could be found. This approach represents a method for statistically controlling for all the potential confounding variables available in the data set.

In addition to these primary analyses, it was determined whether these effects translated across surgical method (open and minimally invasive); and whether the impact of list order varied according to procedure complexity. The procedures were separated by classifying them as those performed using open or minimally invasive techniques to address the first question, and by complexity for the second question, and the matched analysis repeated.

To provide a measure of the magnitude (or effect size) of the analysed variables on list order, the change in the log scale for the linear mixed‐effects model was reported, along with mean difference in the log duration of the procedures in the matched analysis. Change in log duration is, to a high degree of approximation, the geometric average of the proportional percentage change in procedure duration; the percentage change is therefore referred to for all outcomes to provide an intuitive means of understanding these data.

## Results

**Figure 2:**
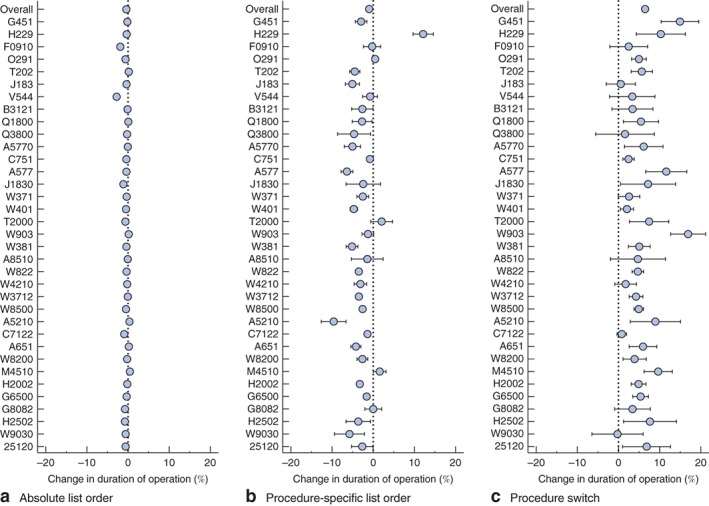
Forest plots showing percentage change in duration of operation for the top 35 procedures in the database based on the influence of fixed model parameters: **a** absolute list order, **b** procedure‐specific list order and **c** procedure switch. Negative values indicate the percentage reduction in duration of operation given an increase in each parameter, and positive values the percentage increase. The top row in each panel shows the overall effect of each fixed parameter. Error bars represent 95 per cent confidence intervals. Procedures are identified by AXA Specialist Procedure Codes (Table S1, supporting information)

Surgical lists containing the 35 operations performed most frequently between 1 April 2013 and 31 May 2015 were analysed (255 757 procedures). The linear mixed‐effects model revealed statistically reliable differences in changes in duration of operation for the fixed effects of absolute list order, procedure‐specific list order and switching when pooled across operations (all P < 0·001). The effect sizes (which can be treated as percentage changes in operating time as a function of list position change) were largest for procedure‐specific list order and switching. The percentage change in duration of operation for each procedure is shown in Fig.  2.

**Figure 3:**
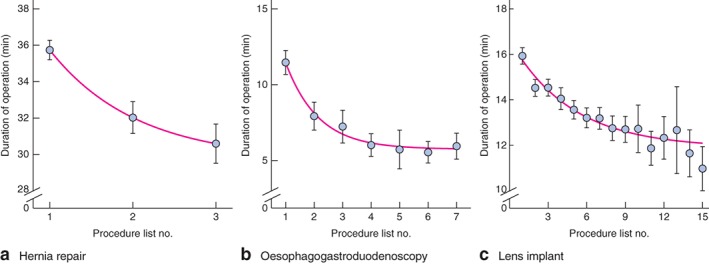
Duration of operation as a function of procedure‐specific list order for three routine procedures: **a** primary open inguinal hernia repair (with mesh); **b** oesophagogastroduodenoscopy (with biopsy of lesion) and **c** lens implant for cataract. Error bars represent 95 per cent confidence intervals

For absolute list order, there was a statistically significant list order effect, suggesting that each position in the list decreases duration of operation by 0·39 (95 per cent c.i. 0·35 to 0·44) per cent across all operations. These effects were substantially greater when considering the benefits acrued when the same procedure was repeated in a list, with the effect of procedure‐specific list order leading to a 0·98 (0·88 to 1·09) per cent reduction in duration. There was a cost associated with switching between different procedures in a list, leading to an increase in duration of operation by 6·48 (6·05 to 6·90) per cent for each increase in position in list order. To illustrate this effect on individual procedures, *Fig*.  3 shows the influence of task repetition on operating time for three routine procedures. There was a marked similarity in the pattern of results across these procedures, indicating that fatigue, inattention and monotony‐related performance impairment following multiple repetitions of a procedure were not present in these data.

Using the same linear model to analyse duration of hospital stay, overall a statistically reliable effect of absolute and procedure‐specific list order was found (both *P* < 0·001), but not for switching (*P* = 0·136). Specifically, the data indicated that, for every increase in absolute list position, duration of hospital stay increased by 0·55 (95 per cent c.i. 0·50 to 0·61) per cent. However, procedure‐specific list order resulted in a decrease in length of stay by 0·72 (0·58 to 0·85) per cent.

**Figure 4:**
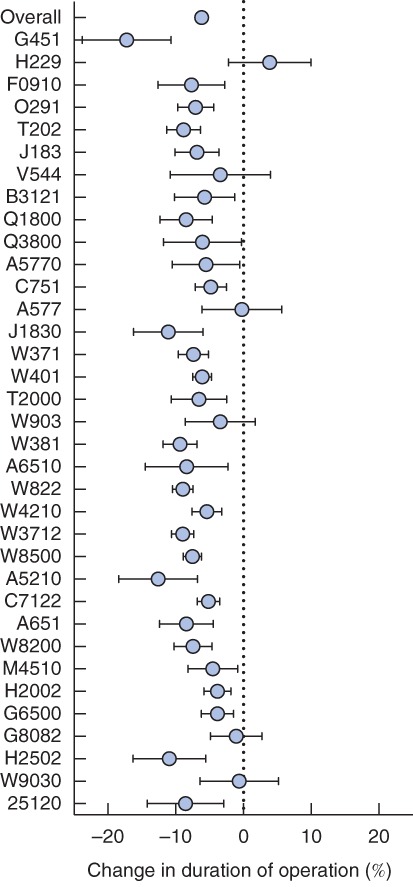
Forest plot from matched analysis illustrating the percentage change in duration of operation for procedure‐specific list order 2 procedures compared with list order 1. Error bars represent 95 per cent confidence intervals. Procedures are identified by AXA Specialist Procedure Codes (Table S1, supporting information)

The matched analysis allowed a focus on the impact of repeating a procedure in more detail on the primary outcome measure of duration of operation. A total of 48 632 pairs were matched from of a maximum 48 699 cases (99·9 per cent of all cases; the sample size was constrained by the number of cases with a procedure‐specific list order of 2 in the data set). Here, a statistically reliable improvement was found (*P* < 0·050) in 29 of the 35 procedures; the change in operating time ranged from a reduction of 3·84 (95 per cent c.i. 1·47 to 6·21) per cent to 17·25 (10·69 to 23·81) per cent (*Fig*.  4). Pooling across all 35 procedures showed a 6·18 (5·64 to 6·72) per cent reduction in operating time on average when performing the second procedure relative to the first (*P* < 0·001).

**Figure 5:**
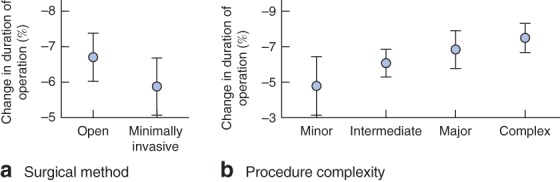
Percentage change in duration of operation time according to **a** surgical method and **b** complexity for matched wake analysis. Error bars represent 95 per cent confidence intervals

Supplementary analyses allowed these results to be assessed in more detail. Conducting the matched analysis separately for open and minimally invasive procedures showed comparable effects of list order on duration of operation, indicating that this phenomenon transcends surgical method (*Fig*.  5 *a*).

Finally, by separating procedures based on their complexity, a weak positive trend was found, but overall there were no differences in effect size as a function of complexity, with the reduction in operating time ranging from 4·80 to 7·50 per cent (*Fig*.  5 *b*).

## Discussion

These results support the suggestion that areas where surgical performance can be improved can be identified through analysis of routinely collected large data sets. The order in which surgical procedures are done has a relationship with their duration. The effects were similar for open and minimally invasive procedures, and procedures of differing complexity. In contrast, switching between different procedures resulted in increased duration of operation. These changes in operating time are particularly significant given that they were observed in highly trained individuals with several years of practice. The results are all the more remarkable when the wide range of factors that can potentially influence procedure duration is considered. The consistency of this pattern of results across procedure type, method and complexity provides compelling evidence that operating list order plays an important role in surgical performance.

The effects of list order on duration of hospital stay are more difficult to interpret. A reduction in length of stay demonstrated in some investigations, with an increase in duration demonstrated in others, brings into question the practical significance of these findings. They are likely to reflect the complex, multifactorial nature of duration of hospital stay, which is affected to a much greater degree by social and institutional factors than operating time. Consequently, efforts were focused on understanding the effects of list order on duration of operation in the follow‐up matched analysis.

The data have practical implications. There was an overall 6·18 per cent saving in operating time (as large as 17·25 per cent in some procedures) for repeating the same procedure on the list, even after controlling for age and ASA grade. This control is particularly important as anecdotal evidence indicates that surgeons typically take these factors into account when compiling their lists, but the data indicate that the process of list ordering itself influences the duration of operation above and beyond the variance captured by age and ASA grade.

From the perspective of service delivery, the results indicate that lists involving a combination of procedures take longer to complete than those that include only one procedure type (the overall cost of switching was estimated as a 6·48 per cent increase in duration). Although increased time with task switching has long been established in experimental psychology^48^, this is the first demonstration of its influence in surgical performance. Where possible, theatre lists should be confined to a single procedure type and method.

One limitation of the present analyses is that the data cannot address the issue of the mechanisms of performance facilitation. However, the results do triangulate with existing empirical work showing that warm up reduces operating times and task switching increases completion times^25^. This indicates a need to explore these areas through further research. An understanding of the optimal preparatory routines to drive performance improvement will be necessary to harness the potential of the phenomenon reported here.

The present data were derived from a private healthcare provider. It is worth noting that the majority of UK healthcare delivery is provided by the NHS, but the private sector is used by 10–22 per cent of the population (depending on region)^49^, ^50^. This data set was chosen for two reasons. First, the data could be pooled across multiple hospitals (a considerable logistical challenge in the NHS)^51^. Second, all operations performed in private UK hospitals must be conducted by trained consultant surgeons, eliminating training effects and ensuring that all procedures on a theatre list were performed by a single practitioner. Further work is required to establish whether these effects also exist in the NHS, where surgeons performing the procedures vary in experience. Environmental factors such as distractions, ward rounds and general resource constraints and prioritization also differ between private and NHS hospitals.

The effects reported here are modest. Yet, it is evident that the aggregation of even small gains has the potential to produce substantial benefits when scaled across a health service; this is particularly important given the growing economic pressures to optimize elective surgery^10^. Although the full extent of the impact of these effects remains to be seen, to put the present results in context, 52 per cent of the procedures analysed involved switching from the preceding operation, and the present results demonstrate that switching is responsible for a 6·48 per cent increase in duration of operation. Over the course of a year in a typical large hospital, avoiding switching could lead to a meaningful reduction in operating time.

## Editor's comments

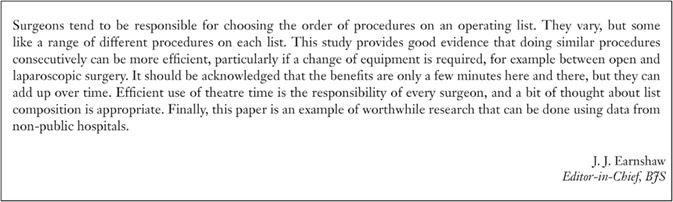

## Supporting information


**Table S1** Procedure code modality and difficulty classification
