# Supplementary material for: Operating list composition and surgical performance
Source: Br J Surg. 2018 Mar 20;105(8):1061–9. doi: 10.1002/bjs.10804 (PMC6032881; doi:10.1002/bjs.10804)
Supplement: Supplementary file 1 — Table S1 Procedure code modality and difficulty classification [file BJS-105-1061-s001.docx]

**BJS10804**

**Operating list composition and surgical performance**

T. W. Pike, F. Mushtaq, R. Mann, P. Chambers, G. Hall, J. Tomlinson, R. Mir, R. M. Wilkie, M. Mon-Williams and J. P. A. Lodge

**Table S1** Procedure code modality and difficulty classification

| Procedure Code | Procedure Description | Modality | Difficulty |
| --- | --- | --- | --- |
| G451 | OGD (with biopsy of lesion) | MIS | Intermediate |
| H229 | Endoscopic Examination of Colon (Unspecified, Diagnostic) | MIS | Intermediate |
| F0910 | Extraction of Impacted/Buried Tooth/Teeth | Open | Intermediate |
| O291 | Subacromial Decompression | Open | Intermediate |
| T202 | Primary Inguinal Hernia Repair (with mesh) | Open | Intermediate |
| J183 | Cholecystectomy | Open | Complex |
| V544 | Spinal Injection | NA | Intermediate |
| B3121 | Bilateral Augmentation Mammoplasty | Open | Intermediate |
| Q1800 | Hysteroscopy | MIS | Intermediate |
| Q3800 | Laparoscopy and Therapeutic Procedure (Gynecological) | MIS | Major |
| A5770 | Facet Joint Injection | NA | Intermediate |
| C751 | Lens Implant | Open | Major |
| A577 | Injection Around Spinal Nerve Root (Therapeutic) | NA | Intermediate |
| J1830 | Laparoscopic Cholecystectomy | MIS | Complex |
| W371 | Primary Total Hip Replacement with Cement | Open | Complex |
| W401 | Primary Total Knee Replacement (with cement) | Open | Complex |
| T2000 | Primary Inguinal Hernia Repair | Open | Intermediate |
| W903 | Joint injection (Therapeutic) | NA | Minor |
| W381 | Primary Total Hip Replacement (without cement) | Open | Complex |
| A6510 | Endoscopic Carpel Tunnel Release | MIS | Intermediate |
| W822 | Endoscopic Resection of Semilunar Cartilage | MIS | Intermediate |
| W4210 | Total Knee Replacement +/- Cement | Open | Complex |
| W3712 | Primary Total Hip Replacement +/- Cement | Open | Complex |
| W8500 | Knee Arthroscopy (multiple) | MIS | Major |
| A5210 | Epidural Injection | NA | Minor |
| C7122 | Phakoemulsification of Lens with Implant | Open | Intermediate |
| A651 | Carpal Tunnel Release | Open | Intermediate |
| W8200 | Arthroscopic Meniscectomy | MIS | Major |
| M4510 | Cystoscopy (Diagnostic) | MIS | Minor |
| H2002 | Colonoscopy (Diagnostic) | MIS | Intermediate |
| G6500 | OGD (Diagnostic) | MIS | Minor |
| G8082 | OGD + Colonoscopy | MIS | Intermediate |
| H2502 | Flexible Sigmoidoscopy (Diagnostic) | MIS | Minor |
| W9030 | Joint Injection (with image guidance) | NA | Minor |
| 25120 | Dorsal Root Ganglion Block | NA | Intermediate |
